# Policy in undergraduate physiotherapy clinical education: Lessons from the implementation of the framework and strategy for disability and rehabilitation

**DOI:** 10.4102/sajp.v82i2.2317

**Published:** 2026-04-30

**Authors:** Naeema A.R. Hussein El Kout, Sonti I. Pilusa, Natalie Benjamin-Damons

**Affiliations:** 1 Department of Physiotherapy, School of Therapeutic Sciences, Faculty of Health Sciences, University of the Witwatersrand, Johannesburg, South Africa

**Keywords:** physiotherapy education, clinical education, disability, rehabilitation, health policy, implementation science, South Africa

## Abstract

**Background:**

Physiotherapy clinical education in South Africa faces persistent challenges related to equity, relevance and sustainability, compounded by resource constraints and workforce pressures. Policy, as a tool for shaping undergraduate clinical training, remains underutilised despite its potential to drive transformative educational practices.

**Objectives:**

This study explores how policy can inform and strengthen undergraduate physiotherapy clinical education, drawing on findings from a doctoral evaluation of the Framework and Strategy for Disability and Rehabilitation (FSDR) in South Africa.

**Method:**

An explanatory mixed-methods design was employed, guided by implementation science frameworks, to evaluate barriers, enablers and outcomes of FSDR implementation. Data were analysed to identify implications for clinical education, including curriculum design, clinical placements and supervisory practices.

**Results:**

Key barriers identified included limited funding, insufficient resources, weak intersectoral collaboration and workforce shortages. Enablers included active disability advocacy, alignment with international rights-based frameworks and community-based rehabilitation initiatives. Applying these findings to clinical education highlighted strategies for optimising clinical placement resources, building supervisor capacity, integrating disability-inclusive and community-based approaches and developing robust monitoring and evaluation systems to ensure training is relevant and socially accountable.

**Conclusion:**

Policy-driven transformation of physiotherapy clinical education is essential to equip graduates with the competencies required to meet societal and health system needs.

**Clinical implication:**

Embedding policy into clinical training fosters socially accountable graduates who can advance disability rights, contribute to equitable rehabilitation services and translate national health priorities into practical clinical practice.

## Introduction

Physiotherapy occupies a central role in addressing functional limitations, preventing disability and promoting participation across the lifespan. In South Africa, the profession is confronted by complex challenges: a growing burden of disability, high prevalence of non-communicable and communicable diseases, entrenched inequities in access to care and an under-resourced public health system. These systemic pressures are mirrored in physiotherapy education, particularly in the domain of clinical education, which constitutes the cornerstone of undergraduate training and professional competence (Newstead et al. 2019).

Clinical education is more than a pedagogical exercise; it represents the nexus between health systems and higher education. Students’ learning experiences are shaped not only by supervisors and curricula but also by the policy and resource environment of clinical sites. In South Africa, this nexus has historically been skewed towards urban, hospital-based settings, privileging biomedical models of care while offering limited exposure to community-based rehabilitation, disability-inclusive practice or intersectoral approaches (Morris et al. 2021). Such misalignment between training and health system needs undermines the preparation of graduates for the realities of practice in resource-constrained contexts.

Clinical education also reflects broader social inequities in South Africa. Students often rotate through urban hospitals with relatively more resources, while rural placements remain under-supported. This imbalance not only disadvantages students in gaining exposure to diverse contexts but also perpetuates inequities in service provision. Graduates may emerge less prepared to deliver care in underserved communities, reinforcing cycles of exclusion for persons with disabilities.

Furthermore, clinical education does not exist in isolation from policy shifts such as National Health Insurance (NHI). The NHI envisages an equitable, primary health care-driven system, yet current physiotherapy education largely trains graduates for hospital-centric practice. A policy-informed shift is needed to prepare students for the preventive, rehabilitative and community-oriented roles envisaged under NHI.

Finally, clinical education represents a site of professional identity formation. Embedding disability rights, equity and policy literacy into training can cultivate a new generation of physiotherapists who view themselves not only as clinicians but also as advocates and policy actors. Such transformation requires intentional alignment of curricula with policy frameworks such as the Framework and Strategy for Disability and Rehabilitation (FSDR) and White Paper on the Rights of Persons with Disabilities (WPRPD).

Policy has the potential to act as a transformative lever for physiotherapy education. Policies articulate rights, set strategic directions and allocate resources that shape service delivery. Yet the role of policy in physiotherapy education is often indirect, fragmented or under-theorised. For instance, the Health Professions Council of South Africa (HPCSA) prescribes core competencies and professional outcomes, but little attention has been paid to how broader health and disability policies such as the WPRPD or the FSDR can be harnessed to reshape curricula and clinical education practices.

The doctoral research on which this article draws evaluated the implementation outcomes of the FSDR in Gauteng province using an explanatory mixed-methods approach (Hussein El Kout 2025). The FSDR was introduced to align South Africa with global disability rights frameworks, particularly the UN Convention on the Rights of Persons with Disabilities (UNCRPD), and to strengthen rehabilitation services through equitable, community-oriented strategies. Its evaluation revealed systemic barriers but also identified promising enabling factors and evidence-based strategies to enhance policy implementation.

This article reframes those findings to address a critical question: *How can disability and rehabilitation policy inform the transformation of undergraduate physiotherapy clinical education in South Africa?* By drawing on the FSDR’s implementation outcomes, we argue that policy-informed implementation strategies can advance undergraduate physiotherapy education by aligning clinical training with health system needs, embedding disability-inclusive and community-based approaches and strengthening intersectoral collaboration. In doing so, physiotherapy education can contribute to broader health system transformation and the advancement of disability rights.

Despite the centrality of policy in shaping service delivery, its integration into professional curricula remains fragmented. International studies highlight that when policies are embedded in health education, students develop stronger competencies in advocacy, systems thinking and social accountability (Frenk et al. 2010). South Africa, however, has not systematically leveraged disability and rehabilitation policy in this way.

The global Rehabilitation 2030 agenda explicitly calls for the training of health professionals to meet growing rehabilitation needs. This includes incorporating rehabilitation into all levels of health systems and ensuring professional curricula reflect community-based and rights-oriented approaches. South Africa’s physiotherapy education can align with this global agenda by embedding FSDR-derived strategies into undergraduate training.

Moreover, South Africa’s broader higher education transformation discourse emphasises decolonisation and responsiveness to local contexts. Policy-informed clinical education provides a pathway to decolonise curricula by shifting focus from Eurocentric, hospital-based models towards contextually relevant, community-based rehabilitation that addresses the lived realities of persons with disabilities.

Clinical education constitutes approximately 50% of undergraduate physiotherapy training in South Africa (Naidoo et al. 2018). It is during clinical placements that students translate theoretical knowledge into practice, develop clinical reasoning and acquire professional identity. Clinical training sites include hospitals, primary healthcare clinics and community-based settings, yet the distribution and quality of these sites vary considerably (Louw et al. 2023).

Challenges affecting clinical education in physiotherapy in South Africa are multifaceted. Inequitable access to clinical sites remains a significant barrier, as training is predominantly concentrated in urban, tertiary hospitals, limiting students’ exposure to rural and community contexts where many persons with disabilities live (Amosun, Jelsma, & Maart 2019; Hussein El Kout, Pilusa & Masuku 2022; Mkabile & Swartz 2020). Resource shortages further compound these challenges, with many facilities lacking essential equipment, adequate supervision and the infrastructure necessary to support effective student learning (McIntyre, Cleland & Ramklass 2021; Morris et al. 2021). Workforce constraints also impede clinical education, as clinical educators and supervisors are often stretched between service delivery and teaching responsibilities, frequently without formal training in educational methods (Caves, Baumann & Renold 2019; Philpott et al. 2020; Tiwari, Ned & Chikte 2020). Policy–practice gaps are evident, as curricula, although aligned with international accreditation standards, inadequately reflect local health priorities, including disability-inclusive care, interprofessional collaboration and primary health care approaches (Banks et al. 2021; Hussein El Kout, Pilusa & Benjamin-Damons 2023; Wu et al. 2020). Moreover, the implementation of national frameworks and strategies for disability and rehabilitation services has been inconsistent, highlighting the need for integration of policy into clinical education to ensure alignment between teaching, service delivery and local community needs (Philpott et al. 2020; Trani et al. 2020). Aligning clinical education with principles of equity and inclusion is essential to operationalise South Africa’s commitments under the UN Convention on the Rights of Persons with Disabilities (UNCRPD), ensuring that graduates are capable of providing culturally sensitive, accessible and community-centred rehabilitation services (Banks et al. 2021; Hussein El Kout et al. 2022). Transformation of clinical education is therefore imperative to ensure that physiotherapy graduates are equipped to meet the complex demands of South Africa’s health system and contribute to equitable access to rehabilitation services, ultimately strengthening the country’s capacity to deliver on disability rights and inclusive health policy objectives (McIntyre et al. 2021; Trani et al. 2020).

Policies are critical instruments for shaping health professional education, serving as drivers for transformation in curricula and training environments. Globally, initiatives such as the World Health Organization’s Rehabilitation 2030 Call to Action and the Sustainable Development Goals emphasise the need for health professional training that is responsive, equitable and community-oriented (Banks et al. 2021; Philpott et al. 2020). In South Africa, policy frameworks, including the National Development Plan 2030, the NHI framework and the FSDR, articulate goals for inclusive, rights-based healthcare, highlighting the importance of aligning professional education with principles of social accountability, equity and disability rights (Hussein El Kout et al. 2022, 2023; Trani et al. 2020).

Policy shapes clinical education in multiple ways, including determining funding and resourcing of health facilities where students are trained, defining the scope of practice and competencies expected of graduates, setting national priorities such as community-based care and disability inclusion that should be reflected in curricula and providing mechanisms for monitoring and evaluation of training outcomes (McIntyre et al. 2021; Morris et al. 2021; Philpott et al. 2020). Despite the existence of these policies, their integration into physiotherapy curricula, particularly within clinical education, remains limited and has not been systematically examined (Banks et al. 2021; Hussein El Kout et al. 2023).

The FSDR (2015–2020, extended to 2023) represented South Africa’s primary policy instrument for strengthening rehabilitation services. It sought to align national efforts with the UN Convention on the Rights of Persons with Disabilities (UNCRPD), advance community-based rehabilitation and ensure equitable access to services (Hussein El Kout et al. 2022; Trani et al. 2020). Evaluation of the FSDR revealed systemic barriers, including inadequate funding, insufficient rehabilitation personnel, weak intersectoral collaboration, inefficiencies in referral pathways and lack of standardised training, alongside enablers such as engagement of advocacy organisations, alignment with global disability rights and active community-based initiatives. Strategies proposed to optimise resource allocation, build workforce capacity, promote intersectoral collaboration and implement comprehensive monitoring and evaluation systems have direct relevance for physiotherapy education, indicating opportunities to embed policy-driven strategies into clinical training environments (Banks et al. 2021; Hussein El Kout et al. 2023).

Implementation science provides a valuable lens for understanding how evidence-based policies and interventions are adopted, adapted and sustained in real-world contexts, offering structured approaches to identify barriers, facilitators and strategies for embedding change in education (McIntyre et al. 2021; Philpott et al. 2020). Frameworks such as the Consolidated Framework for Implementation Research (CFIR), the Exploration, Preparation, Implementation and Sustainment (EPIS) model and the Proctor model of implementation outcomes have been applied in health services research and are particularly relevant to clinical education. Key implementation outcomes applicable to physiotherapy training include acceptability or whether curricula and clinical placements are perceived as relevant and appropriate by students, educators and facilities; adoption, reflecting the extent to which policy-driven practices such as community-based rehabilitation are incorporated into training; feasibility, considering whether clinical sites can realistically support policy-informed training given resource constraints; and sustainability, assessing whether policy-aligned educational practices are maintained over time (Banks et al. 2021; Hussein El Kout et al. 2022; Philpott et al. 2020). Applying an implementation science lens thus allows for systematic examination of how strategies derived from the FSDR can be integrated into undergraduate physiotherapy clinical education to strengthen equity, inclusion and policy alignment.

## Research methods and design

### Study design

This article draws on a secondary analysis of findings from a doctoral thesis titled ‘Evaluation of the Implementation Outcomes of the Framework and Strategy for Disability and Rehabilitation for South Africa: An Explanatory Mixed-Methods Study’ (Hussein El Kout 2025). The original study employed an explanatory sequential mixed-methods design to evaluate the implementation outcomes of the FSDR in Gauteng province.

Secondary analysis was undertaken through an interpretive, conceptual translation of FSDR findings to physiotherapy education. This aligns with approaches in health professions education research where policy analysis informs curricular innovation. The analytical process was iterative, involving mapping barriers and strategies from the FSDR evaluation onto identified gaps in physiotherapy clinical education.

Implementation science provided the scaffolding for this translation. For instance, Proctor’s (Proctor et al. 2011) outcomes of feasibility, adoption and sustainability were used to assess how FSDR strategies could realistically be applied in training contexts. The EPIS framework helped situate findings within the broader EPIS cycle relevant to educational reform.

To enhance trustworthiness, triangulation was achieved by drawing on multiple sources: the original thesis findings, published articles arising from the study and the broader literature on physiotherapy education and policy. This ensured that the proposed strategies were grounded both in empirical evidence and in theoretical frameworks.

In this article, findings are reanalysed and reframed to address the role of policy in physiotherapy clinical education. The approach involves interpretive application of the FSDR implementation strategies to the domain of undergraduate education, guided by implementation science frameworks.

### Data sources

The original study utilised:

- Document review: Provincial and national reports on FSDR implementation.
- Qualitative interviews: Semi-structured interviews with rehabilitation professionals, policymakers and community representatives.
- Focus group discussions (FGDs): Engaging rehabilitation service providers and advocacy groups.

For this article, these data sources are used as an evidence base to draw parallels between policy implementation in rehabilitation services and challenges in physiotherapy clinical education.

### Analytical framework

The analysis applied the Proctor model (Proctor et al. 2011), the EPIS framework and the CFIR to identify implementation outcomes and strategies relevant to physiotherapy education. The adaptation process involved:

- Identifying FSDR implementation outcomes (barriers, facilitators and strategies).
- Mapping these outcomes onto challenges and opportunities in physiotherapy clinical education.
- Developing a conceptual model for policy-informed transformation of clinical education.

### Ethical considerations

Ethical clearance to conduct this study was obtained from the Human and Research Ethics Committee (Medical) of the University of the Witwatersrand (reference number: M220364). Secondary analysis for this article is interpretive and does not involve new data collection; it aligns with ethical principles of academic integrity, responsible data use and contextual sensitivity.

## Results

The reframing of FSDR findings revealed four domains of relevance to physiotherapy clinical education: resource allocation, workforce capacity, intersectoral collaboration, and monitoring and evaluation. The analysis demonstrated that resource allocation in clinical education is not only about physical infrastructure but also about equitable distribution of learning opportunities. Students placed in resource-poor clinics often face demotivation and limited learning experiences, underscoring the need for policy-directed investments that ensure parity across training sites. In terms of workforce capacity, findings highlighted that supervision is inconsistently valued or recognised within health systems. Many clinicians see teaching as an added burden rather than an integral professional responsibility. Policy can provide incentives, recognition and structured pathways for clinician-educators, thereby embedding supervision as a valued role. With respect to monitoring and evaluation, the application of implementation science outcomes illustrates how clinical education can be tracked more systematically. For example, penetration could measure how many students rotate through community-based sites; adoption could assess how many institutions incorporate disability-inclusive training; and sustainability could evaluate the persistence of these practices over time.

### Resource allocation and infrastructure

Framework and Strategy for Disability and Rehabilitation Findings: Implementation was hampered by inadequate funding, limited rehabilitation infrastructure and inequitable resource distribution.

Application to physiotherapy education: Clinical education similarly suffers from resource shortages, including insufficient training sites, lack of equipment and inadequate student support systems. Policy can guide equitable allocation of resources to rural and underserved areas, ensuring that students gain exposure to diverse clinical contexts. For example, structured funding for community-based training sites could expand learning opportunities beyond tertiary hospitals.

### Workforce capacity and supervision

Framework and Strategy for Disability and Rehabilitation Findings: Shortages of skilled rehabilitation personnel constrained service delivery. Lack of standardised training further limited effective implementation.

Application to physiotherapy education: Clinical supervision is undermined by limited numbers of trained supervisors, heavy service delivery demands and inconsistent supervisory practices. Policy-driven strategies, such as capacity-building programmes for clinical educators and standardised supervisor training, can strengthen the quality and consistency of student learning. Embedding disability-inclusive training across curricula ensures that students graduate with competencies aligned with policy goals.

### Intersectoral collaboration and partnerships

Framework and Strategy for Disability and Rehabilitation Findings: Poor collaboration between health, education and social sectors undermined FSDR implementation. However, advocacy organisations and community-based rehabilitation initiatives played enabling roles.

Application to physiotherapy education: Clinical education often occurs in isolation from broader community and advocacy structures. Policy can facilitate partnerships between universities, health departments, disability organisations and communities to create richer, more socially accountable training environments. Interprofessional education (IPE) models could further enhance collaboration and responsiveness to complex health needs.

#### Monitoring, evaluation and accountability

Framework and Strategy for Disability and Rehabilitation Findings: Weak monitoring and evaluation mechanisms limited the ability to track progress and adapt strategies.

#### Application to physiotherapy education

Clinical education lacks thorough mechanisms for evaluating outcomes beyond student assessments. Policy-driven monitoring systems can track indicators such as accessibility, appropriateness and sustainability of clinical training. Aligning evaluation metrics with implementation science outcomes (e.g. penetration, adoption and feasibility) ensures accountability and continuous improvement.

## Discussion

The reframed analysis demonstrates that policy is not merely a backdrop to physiotherapy education but functions as a transformative lever. By embedding policy-driven strategies into curricula and clinical education, universities can better align training with health system needs, advance disability rights and prepare graduates for socially accountable practice (Banks et al. 2021; Hussein El Kout et al. 2022; Philpott et al. 2020). Drawing on findings from the doctoral study, four interrelated implementation strategies are proposed to operationalise policy-informed educational transformation. Firstly, optimising resource allocation for clinical education requires establishing targeted funding for rural and community-based training sites, ensuring equitable distribution of equipment and infrastructure to support student learning and integrating clinical placements into NHI service delivery planning (McIntyre et al. 2021; Morris et al. 2021). Secondly, building educator and supervisor capacity involves developing structured training programmes in supervision and disability-inclusive education, providing protected time and incentives for clinicians to engage in supervision and standardising supervisory practices across institutions (Caves et al. 2019; Philpott et al. 2020). Thirdly, promoting intersectoral collaboration entails forging partnerships between universities, health departments, non-governmental organisations and disabled persons’ organisations (DPOs), embedding students in community-based rehabilitation programmes to enhance contextual learning and expanding IPE initiatives (Banks et al. 2021; Trani et al. 2020). Fourthly, developing thorough monitoring and evaluation systems requires implementing indicators aligned with implementation science outcomes to track clinical education quality, establishing feedback mechanisms involving students, supervisors and communities and integrating evaluation into national health and education policy processes (Hussein El Kout et al. 2023; Philpott et al. 2020).

Embedding policy-informed strategies has profound implications for curriculum reform. Disability-inclusive education should be mainstreamed across modules rather than confined to electives, while community-based placements should be mandatory, structured and well-supported (Banks et al. 2021; Hussein El Kout et al. 2022). Policy literacy must be cultivated as a core graduate competency, equipping physiotherapists to critically engage with health systems and advocate for equitable rehabilitation services.

Students who understand how health policies are formulated, implemented and evaluated are better positioned to serve as advocates and leaders in their profession. Embedding policy education within clinical placements provides real-world opportunities for students to witness the direct impact of policy on patient care (McIntyre et al. 2021; Philpott et al. 2020). Furthermore, clinical education itself is a site of policy implementation; hospitals and clinics where students are placed are often the frontlines where national policies such as the FSDR or NHI are enacted; and aligning student training with these realities enhances relevance and prepares graduates for policy translation in practice (Hussein El Kout et al. 2023; Trani et al. 2020).

The discussion also highlights the potential for policy-informed IPE.

Rehabilitation policies emphasise team-based care, yet physiotherapy education often operates in silos.

Applying FSDR strategies to promote intersectoral collaboration can catalyse more meaningful IPE, strengthening teamwork and holistic patient care (Banks et al. 2021; Philpott et al. 2020). For universities, recommendations extend beyond curricular revision to creating policy-embedded learning environments where students actively engage with disability rights organisations, policymakers and communities, which could be formalised through service-learning modules.

For government, beyond funding, developing policy-education alignment platforms, such as joint committees between health and higher education sectors, can ensure that clinical education policies mirror national health priorities. Professional associations should advocate for policy-informed continuing professional development (CPD) programmes for clinicians, ensuring alignment across the continuum of education and practice (Hussein El Kout et al. 2022; McIntyre et al. 2021). Communities and DPOs should engage as partners in training design and evaluation, providing feedback to maintain responsiveness to disability rights and needs (Banks et al. 2021).

To operationalise policy-driven transformation of physiotherapy clinical education, specific recommendations are proposed for multiple stakeholders. For universities and the HPCSA, accreditation standards should be revised to require integration of disability-inclusive and community-based learning; supervisor training should become a core requirement; and implementation science frameworks should be incorporated into curricula (Caves et al. 2019; Philpott et al. 2020). For government (National Department of Health and Department of Higher Education and Training), funding must be allocated for rural and community-based training infrastructure; clinical education policies should align with NHI and rehabilitation priorities; and joint monitoring systems linking education and service delivery outcomes should be developed (McIntyre et al. 2021; Morris et al. 2021). For professional associations such as the South African Society of Physiotherapy (SASP), advocacy should focus on policy integration in physiotherapy education and CPD support in supervision and policy literacy (Hussein El Kout et al. 2023). Finally, communities and DPOs should engage as partners in the design and evaluation of training programmes and provide feedback to ensure education remains responsive to disability rights and needs (Banks et al. 2021; Trani et al. 2020).

A conceptual policy-informed clinical education implementation model is proposed, adapted from the Proctor model and the EPIS framework. This model emphasises inputs, including policy directives, resources and partnerships; processes such as adoption, feasibility and acceptability; and outcomes, including sustainable, disability-inclusive clinical education aligned with health system needs (Hussein El Kout et al. 2022; Philpott et al. 2020). By applying this model, universities, governments, professional associations and communities can systematically embed policy into physiotherapy clinical education, ensuring graduates are prepared to deliver equitable, socially accountable and rights-based rehabilitation services (Banks et al. 2021; Trani et al. 2020).

## Conclusion

The evidence shows that policy has transformative potential in shaping physiotherapy education. Embedding disability and rehabilitation policy into undergraduate clinical education is not an optional add-on but a necessity if the profession is to remain responsive to societal needs. The adoption of policy-informed implementation strategies ensures that graduates are prepared not only as clinicians but also as advocates, leaders and change agents. This dual role is crucial in advancing equity and upholding the rights of persons with disabilities. Transforming clinical education through policy is about closing the gap between health system needs and educational outputs. In doing so, physiotherapy can contribute meaningfully to South Africa’s vision of an inclusive, equitable and sustainable healthcare system.

Undergraduate physiotherapy clinical education in South Africa is at a crossroads. Entrenched hospital-based models, resource constraints and inequities undermine the preparation of graduates for socially accountable practice. Policy provides a critical yet underutilised lever for transformation. Lessons from the FSDR’s implementation illuminate strategies for optimising resources, building capacity, fostering collaboration and embedding thorough evaluation. By aligning physiotherapy education with policy, South Africa can produce graduates who are not only clinically competent but also agents of health system transformation and disability rights advancement.
